# Validation and Reliability of the Polish Version of the Eating Assessment Tool-10 Questionnaire

**DOI:** 10.3390/nu17081291

**Published:** 2025-04-08

**Authors:** Barbara Jamroz, Magdalena Milewska, Aleksandra Ostrowska, Joanna Chmielewska-Walczak, Mariusz Panczyk, Dorota Szostak-Wegierek

**Affiliations:** 1 Clinical Department of Otolaryngology, National Medical Institute of the Interior and Administration, 02-507 Warsaw, Poland; barbara.jamroz@pimmswia.gov.pl; 2 Department of Clinical Dietetics, Faculty of Health Sciences, Medical University of Warsaw, 02-091 Warsaw, Poland; dorota.szostak-wegierek@wum.edu.pl; 3 Otorhinolaryngology, Head and Neck Surgery Department, Medical University of Warsaw, 02-091 Warsaw, Poland; barjamroz@imid.med.pl (A.O.); joanna.chmielewska@wum.edu.pl (J.C.-W.); 4 Department of Education and Research in Health Sciences, Faculty of Health Sciences, Medical University of Warsaw, 02-091 Warsaw, Poland; mariusz.panczyk@wum.edu.pl

**Keywords:** dysphagia, EAT-10, oropharyngeal dysphagia, screening, validation

## Abstract

**Introduction:** Early identification of patients at risk of dysphagia is of paramount importance. To date, no dysphagia screening questionnaire has been validated and translated into Polish that can be widely used in a multidisciplinary setting. Our study aimed to validate and adapt the Polish version of the Eating Assessment Tool-10 questionnaire (EAT-10). **Materials and Methods:** The EAT-10 questionnaire was translated into Polish using a formal forward–backward translation method. The Polish EAT-10 was administered to 109 patients with different dysphagia etiologies and 24 control subjects. Internal consistency, unidimensionality, test–retest reliability and external validity against the Visual Analog Scale (VAS), and Flexible Endoscopic Evaluation of Swallowing (FEES) were performed. **Results:** The EAT-10PL demonstrated excellent internal consistency (Cronbach’s α = 0.958) and confirmed unidimensionality. We found a strong correlation between EAT-10PL with the Visual Analog Scale (VAS) (rs = 0.94, *p* < 0.001) and a weaker correlation with the PAS (rs = 0.55, *p* < 0.001). We reported a sensitivity of 79.5% and specificity at the level of 60.0%, using ≥3 as a cut-off point. The statistically chosen cutoff point for PAS ≥ 2 and EAT-10 ≥ 6 indicated optimal specificity (70.0%) and sensitivity (79.5%) of measurements performed using EAT-10PL. The EAT-10PL questionnaire demonstrates high discriminatory ability relative to the control group (F(4, 104) = 16.219, *p* < 0.001, η^2^ = 0.38 [95%CI: 0.22–0.48]). **Conclusions:** The Polish EAT-10 is a valid and reliable, self-administered questionnaire for dysphagic patient identification. The Polish EAT-10 ≥ 3 can be considered abnormal; however, it seems that EAT-10PL is not appropriate for patients with dysphagia and a chronic cough background, and further research is required.

## 1. Introduction

Dysphagia is a common symptom connected with stroke and neurological diseases, head and neck cancers, COPD (Chronic Obstructive Pulmonary Diseases) and chronic pulmonary problems, the geriatric population, and many others. The prevalence of swallowing problems is nearly 7% in the general population; it is more common in the elderly population, up to 75% in those having suffered acute stroke, nearly 60% in those with Parkinson’s disease, and 80% in those with dementia [1,2]. Complications of dysphagia can lead to death because of aspiration pneumonia, malnutrition, and dehydration. Dysphagia is also associated with high mortality and morbidity rates and is connected with a poorer quality of life [1,2,3,4,5]. Because of that, many screening tests have been developed to detect patients with objective swallowing problems as the first step in a long, multistage diagnostic procedure, which can provide proper treatment in all cases. Patients with positive screening test results proceed to the next steps—firstly, a clinical evaluation of swallowing and, secondly, instrumental evaluation (FEES (Flexible Endoscopic Evaluation of Swallowing) or/VFSS (Videofluoroscopy Swallowing Study)) [6,7,8]. In everyday practice, it is possible to provide two main types of bedside screening tests: (1) oral trials, like the VVST (volume viscosity swallowing test) or water swallowing test; or (2) with the use of questionnaires, like the SSQ (Sydney Swallow Questionnaire) or EAT-10 (Eating Assessment Tool-10). The Bolus test can evaluate the safety of swallowing, e.g., water swallowing test, Daniel’s test, Blue dye test, or both the safety and efficiency of swallowing, like the VVST, GUSS (Gugging Swallowing Screen), Yale Swallow Protocol, and TOR-BSST (Toronto Bedside Swallow Screening Test) [9,10,11,12,13,14,15,16,17]. All of these have other endpoints and psychometric characteristics. Their sensitivity is between 70 and 100%, and specificity is between 63 and 89% [8]. Sufficient screening tools need to adhere to several criteria: high specificity (≥70%) and sensitivity (≥60%); non-invasive methods; no distress to the patients; an easy, fast, and cheap protocol; reproducible results; and no need for long-term personnel training [8]. The EAT-10 is a short, rapid, self-administrated, validated questionnaire commonly used in clinical practice. It consists of 10 symptom-specific items, using a five-point scale, where 0 means no problem, and 4 indicates the most severe problem. The total score result could be between 0 and 40 points. Three points or more give a suspicion of dysphagia, in which case the patient needs additional clinical examination. Despite its not entirely satisfactory psychometric properties, the EAT-10 questionnaire is a common screening tool [11,18,19,20]. A great advantage of this tool is the possibility of using it in all patient groups, not only in one specific group, e.g., DyMUS (Dysphagia In Multiple Sclerosis) in multiple sclerosis. It can be used for both screening of dysphagia and monitoring of treatment effects. Answers can help in a proper interview, especially if medical personnel (e.g., physicians, SLPs, or dieticians) have little or no experience with dysphagia patients. The EAT-10 questionnaire is not time-consuming, and patients can self-report their answers 5 min before a visit [11]. At present, there is no dysphagia screening questionnaire that has been translated and validated into Polish, which can be used on a wide, multidisciplinary scale. The aim of our study was the validation and adaptation of the Polish EAT-10 questionnaire.

## 2. Materials and Methods

### 2.1. Study Group and Controls

A total of 109 participants (85 dysphagic patients and 24 control patients) were classified in this study from September 2018 to June 2019. Inclusion criteria were dysphagia symptoms, age over 18 years old, state after head and neck surgery, neurological/cardiological/pulmonological diseases, and gastrological diseases. Exclusion criteria were state after nose trauma or nose and paranasal surgery in the last two weeks, coagulation disorders, unconsciousness, no possibility of oral feeding due to reasons other than dysphagia, and not having consented to participate in the study. The control group was 24 healthy adults without dysphagia symptoms or diseases associated with swallowing disorders, who did not take drugs that influenced swallowing physiology. The age of the participants in the study group was similar to the age of those in the control group. The study group was divided into 4 subgroups: head and neck cancer (Group 1), neurological diseases (Group 2), chronic cough (Group 3), and other (Group 4). The main characteristics are presented in Table 1 and Table 2.

### 2.2. Translation and Development of the Polish EAT-10 Questionnaire

The EAT-10 questionnaire was translated into Polish using a formal forward–backward translation method [21]. First, the EAT-10 was translated from English into Polish by two medically trained, bilingual, independent persons, with Polish as their native language. Differences in translation were discussed and resolved by a multidisciplinary team (phoniatrician, SLP, and dietician). The agreed-upon version was returned for back translations to English. The translation was discussed and compared with the original version [11]. The Polish version was pilot tested in 9 patients with diagnosed dysphagia. There were no reported comments or complaints from the patients.

### 2.3. Study Protocol

First step: All the participants completed the Polish version of the EAT 10-PL questionnaire twice over 14 days. Furthermore, the level of severity of their symptoms was indicated on the VAS (Visual Analog Scale), where 0 means no dysphagia symptoms and 10 means the most serious problem.

Second step: Patients and controls had an instrumental evaluation of swallowing (FEES) performed with a Xion nasofiberscope, which was 3.2 mm in diameter and equipped with a light source, camera, and color monitor in accordance with unified study protocol [22]. All participants were examined by a phoniatrician and speech/language pathologist with min. 5 years of experience. Swallowing safety was assessed by two independent specialists using the PAS scale (penetration–aspiration scale) [23]. The PAS score was verified after FEES examination. When the score was not the same, a third researcher (healthcare professional) verified the result. A PAS score of more than 1 was considered abnormal.

FEES examination protocol: each naris was examined visually by a phoniatrician, who then passed a fiberscope through the wider naris, without administration of topic lignocaine anesthesia or vasoconstrictor. We wanted to eliminate potential anesthetic reactions and avoid any impact on the swallowing process. The first part of the examination was evaluation of the anatomy and physiology of the nasopharynx, oropharynx, hypopharynx, and larynx (nasopharyngeal closure during phonation and swallowing, squeeze maneuver, cough efficiency, larynx sensation, saliva swallowing, white-out). Furthermore, all participants received the same food with the same consistency: 5 mL of regular liquid 3 times (non-carbonated water, mixed with 1 drop of green dye, Wilton, series 98, number 610-324/4800, in a ratio of 1:5, color/water), 5 mL of nectar 3 times (200 mL of green-colored water with 1 spoon of Nutilis Clear thickener) and one piece of regular solid food (crackers). For the first sip, oral control was tested (recommendation for the patients: take a sip of water, and do not swallow until I ask); the examination team evaluated the safety and efficiency of swallowing and the presence or absence of penetration, aspiration, residue, premature spillage, and regurgitation. In the third part (only for the study group), an evaluation of adaptation or/and compensation rehabilitation techniques was conducted.

### 2.4. Statistical Analysis

To evaluate the psychometric properties of the EAT-10PL, we assessed the unidimensionality of the scale (principal component analysis); test–retest reliability (intraclass correlation coefficient (ICC)); internal consistency (Cronbach’s alpha); external validity (correlation); and discriminant validity (receiver operating characteristic (ROC) curve).

It was investigated whether floor or ceiling effects took place in the data gathered. In accordance with a study by Terwee et al., it was posited that floor or ceiling effects do not take place if more than 15% of respondents obtain adequate lowest or highest possible scores [24]. An analysis of the unidimensionality of EAT-10PL was performed by means of principal component analysis. It was assumed that a scale can be considered unidimensional when it meets the Kaiser criterion (eigenvalue exceeds value 1 only once), and the degree of recreating the variability of the indicator variables by the first principal component should exceed 40% [25].

The internal consistency of the Polish version of the EAT-10 (EAT-10PL) was assessed using Cronbach’s alpha coefficient, as originally proposed by Cronbach [26]. A threshold value of >0.700 was adopted to indicate satisfactory internal consistency [27]. In addition, item-total correlations were calculated for each item within the scale to examine the extent to which individual items correlated with the total score. According to the guidelines established, these correlations should not fall below 0.200, as lower values may indicate that the item does not contribute meaningfully to the overall construct. In the present analysis, no item-total correlation values below 0.200 were observed, confirming the internal coherence of the scale items.

Separately, the absolute stability of the EAT-10PL was evaluated through a test–retest reliability analysis. This was performed by calculating the intraclass correlation coefficient (ICC), which determines the degree of consistency between responses provided in the first and second administrations of the instrument [28].

External validity was estimated by identifying Spearman’s rank order correlation (rs) between the total EAT-10PL score and the score calculated for the two measurements: VAS and PAS. Known group validity was performed by comparing the mean of ETA-10PL scoring between the control group and the test group. One-way analysis of variance (ANOVA) with Games–Howell Post Hoc Test was used. The effect size was estimated using eta-squared. An ROC curve was used to illustrate the diagnostic ability of the measurement of EAT-10PL. The PAS score (gold standard) with different cut-off points was a binary classifier for building the ROC curve. The optimal cut-point for the EAT-10PL was estimated based on Youden’s index with a 95% confidence interval (bootstrapping) [29]. The sensitivity and specificity of the EAT-10PL measurement were estimated at the optimal cut-off point. Positive predictive value (PPV), negative predictive value (NPV), and accuracy were also determined. Statistical analysis was performed using STATISTICA ver. 13.3 (TIBCO^®^ Software, Palo Alto, CA, USA). A 5% level of significance was set.

## 3. Results

Characteristics of the study group (n = 85) and controls (n = 24) are presented in Table 1. The mean age of the participants was 53.4 ± 16.3 years, ranging from 18 to 80 years. Data on the sex and dysphagia etiology of the participants are reported in Table 1 and Table 2.

### 3.1. Variables

The dysphagic patients had significantly higher total EAT-10PL scores and scored higher on the VAS than controls, *p* < 0.01 and *p* < 0.001, respectively (Table 3).

### 3.2. Item Analysis

The average result obtained for the EAT-10PL in the study sample (N = 85) was 13.6 (SD = 12.39, coefficient of variation = 91.1%) with a minimal value of 0.0 and a maximum value of 40.0. The score was characterized by a right-sided asymmetry (skew = 0.62) and lack of agreement with normal distribution (Shapiro–Wilk test, W = 0.891, *p* < 0.001). For none of the items, zero SD was observed. The score for the majority of the items was characterized by a lack of normal distribution (CR values for skew and kurtosis besides the range [−2, 2]) (Table 4). In 4.7% of the cases in the study sample, the highest possible score was observed (no ceiling effect). However, in 10.6% of the cases in the study sample, the lowest possible score was observed (no floor effect).

### 3.3. Unidimensionality

Using principal component analysis, we verified the eigenvalues and the share of variance explained by the first factor (Kaiser criterion 7.31; 0.65). The share of variance explained by the first principal component was 73.13% For the EAT-10PL scale, two conditions of unidimensionality were fulfilled: only one eigenvalue was noted as being greater than 1.00, and the total variance explained by the first principal component was more than 40%.

### 3.4. Internal Consistency

For the EAT-10PL scale, the obtained value of the Cronbach’s alpha coefficient was 0.958. Correlations among individual items and the total scale score minus the individual items ranged between 0.63 and 0.92 (Table 5). Regardless of the etiology of the disorders, high internal consistency was achieved (head and neck cancer: α = 0.952; neurological diseases: α = 0.908; chronic cough: α = 0.853; and other diseases: α = 0.945).

### 3.5. Test–Retest Reliability

Evaluation of test–retest reliability showed good stability of EAT10PL in the measurement of the study sample (N = 85). Assumptions regarding the stability of the re-measurement results were met. The ranges of ICCs were satisfactory (Table 6). The test–retest analysis for the scale measured by an EAT-10PL was 0.997 ([95%CI: 0.995–0.998], F = 681.440, *p* < 0.001).

### 3.6. External Validity

In terms of the agreement between EAT-10 measurements and the external criterion, a strong correlation was observed with the Visual Analog Scale (VAS) (rs = 0.94, *p* < 0.001), and a slightly weaker correlation was found with the PAS (rs = 0.55, *p* < 0.001).

### 3.7. Known Groups Validation

The Polish version of the EAT-10 questionnaire demonstrates high discriminatory ability relative to the control group (F (4, 104) = 16.219, *p* < 0.001, η^2^ = 0.38) [95%CI: 0.22–0.48]). Post hoc analysis (Games–Howell Post Hoc Test) indicated that only the chronic cough group did not show a statistically significant difference from the control group (Table 7).

### 3.8. Diagnostic Properties

**Figure 1:**
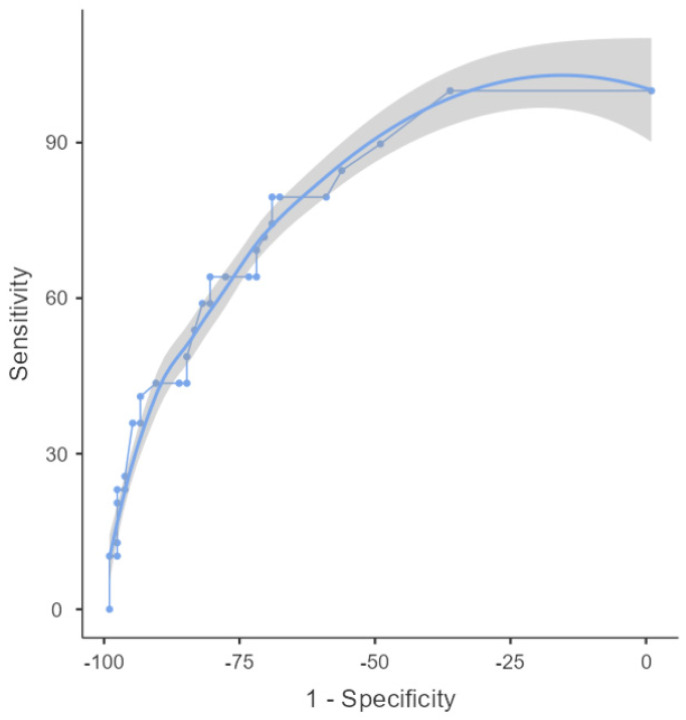
Receiver operating characteristic curve with the performance of a classification model at all classification thresholds. The statistical technique of Locally Weighted Scatterplot Smoothing (LOWESS) was used to make a smooth curve. Gray area—standard error.

The EAT-10PL questionnaire was validated through the analysis of its diagnostic properties, utilizing various cutoff points on the PAS to determine the optimal specificity and sensitivity. The analysis focused on identifying the most effective cutoff point for diagnosing patients using the EAT-10PL questionnaire. The statistically chosen cutoff point for PAS ≥ 2 with an EAT-10PL cutoff point of ≥6 showed balanced sensitivity and specificity, achieving values of 0.795 and 0.700, respectively (Table 8, Figure 1). The comparison between PAS and EAT-10PL measurement results using McNemar’s test suggested a marginally non-significant difference in the measurement results between the two scales at the cutoff point analyzed (Table 9).

## 4. Discussion

Dysphagia is related to a wide range of diseases and affects health, quality of life, and functional capacity. Therefore, early and routine identification of patients at risk of dysphagia is of paramount importance. Although some screening tools are available, few are widely used in clinical practice, especially in Poland. One of the most used tools in clinical practice worldwide is the EAT-10 developed by Belafsky et al. in 2008, which has been translated into several languages [11]. The authors of the recently published systematic review and meta-analysis concluded that, generally, the EAT-10 is characterized by good psychometric properties and internal consistency, confirming previous recommendations of using the EAT-10 as a screening tool. Additionally, a linear correlation between the EAT-10 and PAS score was observed [30]. In our study, the EAT-10 PL was validated against FEES results and the VAS. We found that the Polish EAT-10 Questionnaire demonstrated robust psychometric properties in the assessment of swallowing disorders. The scale showed excellent test–retest reliability, supporting its temporal stability. The internal consistency, as measured by Cronbach’s alpha (α = 0.958), was high and consistent with values reported in other language adaptations of the tool [31,32,33,34,35,36]. While a high alpha coefficient can reflect good internal coherence, it may also indicate potential item redundancy. Several previous studies have raised concerns about overlapping content among certain EAT-10 items and suggested that a more parsimonious version of the scale could be developed without significant loss of information [18,31]. Although we intentionally retained the original 10-item structure to ensure cross-cultural and conceptual equivalence in the validation process, we acknowledge that further investigation—using modern psychometric methods such as item response theory (IRT) or Rasch analysis—could inform future refinements and potential item reduction strategies to optimize the instrument’s efficiency.

Our analysis reported the lowest sensitivity for patients with chronic cough, especially for questions no. 1 (weight loss), no. 9 (cough during meals), and no. 10 (stressful mealtime). We supposed that this low discriminative value resulted from the pathophysiology of chronic cough. Patients usually cough outside of mealtimes. Based on our clinical experience, we did not observe weight loss in this group of patients. The low sensitivity for questions 6 (painful swallowing) and 9 (cough during meals) in patients diagnosed with neurological disease was noticeable. Patients with neurological disease usually present lower sensitivity, which may induce reduced negative predictive value compared to the true results. Schlickewei et al. observed low accuracy of their analyzed tool for patients with Parkinson’s disease [37]. We did not include patients diagnosed with PD, but we included those diagnosed with other neurodegenerative diseases derived (MS, ALS, or MSA), which is characterized by decreased sensitivity of the throat. The differential course of some diseases may affect the sensitivity of some EAT-10 items, which results in lower reliability. Cordier et al. [18] identified some weaknesses in construction validity, including item redundancy. They concluded that items 2, 7, and 10 do not contribute to the general construct and called for redesigning the EAT-10 [18]. Principal component analysis indicated the unidimensionality of the EAT-10 PL, which can suggest that some items do not contribute to the overall model. Our results are in accordance with Cordier et al.’s [18] observations. Contrary to their results, we did not confirm floor effects. Referring to the low sensitivity of some questions in our study, we suppose that with regard to patient diagnostic criteria some of the items may be ill-fitting. Similarly to Plowman and Donohue’s studies, we also obtained excellent discriminatory capacity for dysphagia [38,39]. In our study using the PAS as the reference standard, we reported a sensitivity of 79.5% and specificity at 60%, using ≥3 as a cut-off point. Rofes et al. obtained slightly higher sensitivity and specificity [40]. External validity analysis reported a very high correlation between the EAT-10 and the VAS and a high correlation between the EAT 10 and PAS, which indicated high external validity, similar to the VAS or FEES. The Polish EAT-10 appears to be able to detect the severity of dysphagia. High external validity was found in French, Italian, and Spanish studies [31,41,42]. We showed that impaired safety of swallowing may be highly probable for patients with EAT ≥ 6 points. When we consider PAS ≥ 4 treated as abnormal [23], 28 points potentially indicate the risk of aspiration or aspiration occurrence. Interestingly, analyzing cut-off point for PAS ≥ 6, we discovered 33 points in EAT-10 as the cut-off point for aspiration detection. Other studies reported 16 and 17 points as a cut-off point for better aspiration detection. Shapira-Galitz et al. indicated 8 points as optimal cut-off points for aspiration detection (sensitivity of 87.18% and specificity of 42.55%) [34]. In another study, the sensitivity of an EAT-10 > 15 was 71%, and subjects with an EAT-10 above 15 points were 2.2 times more likely to present aspiration [35]. Discrepancies between results may come from a heterogeneous group of patients and probably by including patients presenting low EAT-10-PL scores. According to the prevalence of aspiration in our study, the positive predictive value was significantly lower (28.95%); however, negative predictive value was higher (94.37%) in comparison with the cited study [42]. Our results show a better sensitivity/specificity balance for the EAT-10-PL ≥ 6 than for EAT-10 ≥ 3, as suggested by Belafsky et al. [11]; however, sensitivity was the same in both analyses. Other authors confirmed a threshold ≥ 3 to distinguish between healthy and dysphagic patients [32,41,42]. Rofes et al. compared EAT 10 results (EAT-10 ≥ 2 cut-off point was applied) with VFSS and reported sensitivity and specificity of 89% and 82%, respectively [39]. We did not set two cut-off points, but a lower cut-off point would probably be associated with a high number of false positives. We should emphasize that, in our use of the EAT-10 as a screening tool, we sought to identify any swallowing problems in patients independently from etiology. We assumed that patients in our study with 13 points and more had a higher probability of having impaired safety of swallowing. However, discrepancies between thresholds may result from group heterogeneity, different severity of swallowing disorders, and different mechanisms underlying impaired safety of swallowing. In this context, it is very difficult to draw any general conclusions. The present study has some limitations. Firstly, the low number of patients reduced its statistical power. Secondly, the heterogeneous population could limit the comparison of some analyses with other studies. We believe that, in validating the EAT-10, both instrumental methods, the FEES as well as VFSS, should be applied to avoid aspiration underestimation. Both methods are characterized by different psychometric properties [43].

## 5. Limitation

This study has several limitations that should be acknowledged. First, although the primary analyses were conducted on the total sample (N = 85), exploratory subgroup analyses (e.g., item–total correlations by diagnostic category) were based on relatively small subsample sizes. As a result, the precision and generalizability of item–level estimates within subgroups—particularly those with low numbers such as the neurological disease group (N = 11)—are limited and should be interpreted with caution.

Second, the study design did not include statistical adjustment for potential covariates (e.g., age, disease duration, cognitive status, or comorbidity burden) that may differ systematically across clinical groups. While the subgroup analyses were not intended for inferential comparison but rather to explore item–level psychometric performance, we recognize that group-level heterogeneity may have influenced the observed variability in item–total correlations.

Future research should address these limitations by employing larger and more diagnostically balanced samples, and by incorporating covariate-adjusted models to more robustly examine differential item functioning and between-group comparisons. Such steps will further strengthen the generalizability and interpretive value of the EAT-10PL across clinical populations.

## 6. Conclusions

The Polish EAT-10 is a valid, reliable, self-administered questionnaire for dysphagic patient identification. Polish EAT-10 scores ≥ 3 can be considered abnormal; however, it seems that the Polish EAT-10 is not appropriate for patients with chronic cough, and further research is required. We recommend using both complementary instrumental methods for aspiration detection (FEES and VFSS) to validate EAT-10.

## Figures and Tables

**Table 1:** Characteristics of the study groups.

	Total (n = 109)	Control Group (n = 24)	Head and Neck Cancer Group 1 (n = 35)	Neurologic Diseases Group 2 (n = 11)	Chronic Cough Group 3 (n = 22)	Other Diseases Group 4 (n = 17)
Age [yrs]						
Mean ± SD	53.4 ± 16.31	48.4 ± 16.16	53.2 ± 16.42	60.5 ± 15.48	57.4 ± 11.93	51.2 ± 20.25
Min.–max.	18–80	24–74	22–80	27–80	34–73	18–80
CV [%]	30.5	33.4	30.9	25.6	20.8	39.6
Gender						
Female [n, %]	66, 60.6%	17, 70.8%	20, 57.1%	2, 18.2%	17, 77.3%	9, 52.9%
Male [n, %]	43, 39.4%	7, 29.2%	15, 42.9%	9, 81.8%	5, 22.7%	8, 47.1%

SD—standard deviation; CV—coefficient of variation.

**Table 2:** The etiology of dysphagia.

**Group 1 (n = 35)**		
	Paraganglioma	n = 12
	Thyroid carcinoma	n = 5
	Cerebellopontine angle tumor	n = 5
	Lingual carcinoma, free flap reconstruction	n = 2
	Larynx carcinoma	n = 3
	Tonsil carcinoma	n = 2
	Pharynx carcinoma	n = 1
	Parapharyngeal space tumor	n = 1
	Cholesteatoma of temporal bone pyramid	n = 1
	Tracheostomy	n = 1
	Brainstem tumor	n = 1
	Aneurysmal of brain vessels	n = 1
**Group 2 (n = 11)**	Stroke	n = 2
	Dystrophia	n = 3
	Myopathy	n = 1
	MS	n = 1
	ALS	n = 2
	MSA	n = 2
**Group 3 (n = 22)**		
	Chronic cough	n = 22
**Group 4 (n = 17)**		
	Reflux disease	n = 9
	Chronic heart disease	n = 5
	Pneumonia	n = 2
	Hepatitis	n = 1

MS—multiple sclerosis; ALS—Amyotrophic Lateral Sclerosis; MSA—multiple system atrophy.

**Table 3:** Screening test results.

	Studied Group (n = 85) M ± SD	Control Group (n = 24) M ± SD	t_df-107_	*p*-Value	MD	95% CI
EAT-10PL	13.60 ± 12.39	0.42 ± 0.72	5.193	<0.001	13.18	8.15; 18.22
VAS	3.01 ± 1.98	0.04 ± 0.20	7.317	<0.001	2.97	2.17; 3.77
PAS	2.69 ± 2.41	1.00 ± 0.00	3.439	0.001	1.69	0.72; 2.67

EAT-10PL—Polish version of the EAT-10 questionnaire; VAS—Visual Analog Scale; PAS—Penetration–Aspiration Scale; M—mean. SD—standard deviation; MD—difference in means; CI—confidence interval.

**Table 4:** Descriptive statistics for ETA-10PL in the study sample (n = 85).

Item	Mean	SD	Skew	CR	Kurtosis	CR
EAT10PL_1	0.92	1.40	0.55	2.052	−1.36	−2.560
EAT10PL_2	1.64	1.71	0.54	2.035	−0.68	−1.276
EAT10PL_3	1.19	1.40	0.50	1.888	−1.24	−2.342
EAT10PL_4	1.69	1.64	0.62	2.333	−1.07	−2.009
EAT10PL_5	1.67	1.63	2.03	7.636	3.14	5.907
EAT10PL_6	0.60	1.11	0.31	1.160	−1.53	−2.885
EAT10PL_7	1.38	1.48	0.29	1.081	−1.51	−2.841
EAT10PL_8	1.54	1.53	0.81	3.050	−0.63	−1.176
EAT10PL_9	1.52	1.27	0.32	1.207	−1.63	−3.063
EAT10PL_10	1.46	1.62	1.30	4.899	0.22	0.415
Total	10.70	12.23	0.953	4.126	−0.372	−0.810

SD—standard deviation; CR—critical ratio (calculated by dividing the empirical statistic by its standard error).

**Table 5:** Item–total correlation analysis.

Item	Item–Total Correlation				
	Study Sample (n = 85)	Head and Neck Cancer (n = 35)	Neurologic Diseases (n = 11)	Chronic Cough (n = 22)	Other Diseases (n = 17)
EAT10PL_1	0.63	0.77	0.61	0.38	0.59
EAT10PL_2	0.69	0.83	0.82	0.53	0.93
EAT10PL_3	0.75	0.78	0.64	0.74	0.70
EAT10PL_4	0.81	0.86	0.91	0.76	0.86
EAT10PL_5	0.83	0.79	0.77	0.53	0.68
EAT10PL_6	0.87	0.64	0.21	0.58	0.65
EAT10PL_7	0.87	0.90	0.81	0.68	0.91
EAT10PL_8	0.90	0.81	0.88	0.76	0.81
EAT10PL_9	0.91	0.72	0.20	0.18	0.87
EAT10PL_10	0.92	0.85	0.98	0.46	0.72

**Table 6:** Test–retest reliability of EAT-10PL.

Item	ICC *	Mean Difference	95%CI for Mean Difference	t_df=68_	*p*-Value **
EAT10PL_1	0.986	0.07	−0.01; 0.14	1.773	0.083
EAT10PL_2	0.985	−0.04	−0.13; 0.05	−1.000	0.323
EAT10PL_3	0.984	−0.02	−0.10; 0.06	−0.573	0.570
EAT10PL_4	0.993	0.00	−0.06; 0.06	0.000	1.000
EAT10PL_5	0.996	0.02	−0.02; 0.07	1.000	0.323
EAT10PL_6	0.979	0.02	−0.06; 0.10	0.573	0.570
EAT10PL_7	0.976	0.09	−0.02; 0.20	1.665	0.103
EAT10PL_8	0.981	−0.07	−0.17; 0.03	−1.354	0.183
EAT10PL_9	0.975	0.02	−0.08; 0.12	0.443	0.660
EAT10PL_10	0.978	0.09	−0.02; 0.20	1.665	0.103
Total	0.996	0.18	−0.19; 0.54	0.984	0.330

ICC—intraclass correlation coefficient; CI—confidence interval. * For ICCs, values more than 0.75 indicated good reliability, and less than 0.75 indicated poor-to-moderate reliability. ** paired *t*-test.

**Table 7:** Comparison of ETA-10PL results between groups.

Comparison			Mean Difference	SE	df	t	*p*-Value *
Head and neck cancer	-	Neurologic diseases	−2.56	3.38	104	−0.757	0.967
	-	Chronic cough	13.30	2.66	104	4.998	<0.001
	-	Other diseases	2.73	2.89	104	0.943	0.949
	-	Control	16.84	2.59	104	6.496	<0.001
Neurological diseases	-	Chronic cough	15.86	3.61	104	4.392	0.006
	-	Other diseases	5.29	3.79	104	1.397	0.774
	-	Control	19.40	3.56	104	5.447	0.001
Chronic cough	-	Other diseases	−10.57	3.16	104	−3.348	0.027
	-	Control	3.54	2.89	104	1.225	0.010
Other diseases	-	Control	14.11	3.1	104	4.551	0.002

* Games–Howell Post Hoc Test.

**Table 8:** Diagnostic properties of the EAT-10PL questionnaire.

PAS Cut-Off Point	EAT-10PL Cut-Off Point	Sens.	Spec.	PPV	NPV	DL(−)	DL(+)	YI	AUC	Accuracy
≥2	≥6	0.795	0.700	0.596	0.860	0.29	2.65	0.495	0.809	0.670
≥3	≥13	0.786	0.719	0.579	0.872	0.30	2.80	0.505	0.799	0.741
≥4	≥28	0.609	0.952	0.824	0.868	0.41	12.58	0.560	0.794	0.859
≥5	≥28	0.619	0.938	0.765	0.882	0.41	9.91	0.557	0.793	0.859
≥6	≥33	0.500	0.959	0.667	0.921	0.52	12.17	0.459	0.711	0.894

Sens.—sensitivity; Spec.—specificity; PPV—positive predictive value; NPV—negative predictive value; DL—disease likelihood ratio; YI—Youden’s index; AUC—area under the curve.

**Table 9:** Comparison of PAS vs. EAT-10PL measurement results.

	EAT-10PL (<6)		EAT-10PL (≥6)		χ^2^_df=1_	*p*-Value *
	N	%	N	%		
PAS < 2	49	44.95	21	19.27	3.613	0.057
PAS ≥ 2	8	7.34	31	28.44		

* McNemar’s test.

## Data Availability

Full documentation is available in the archives of the Central Clinical Hospital of the Medical University of Warsaw, located at 1a Banacha Street, 02-097 Warsaw, Mazowieckie Voivodeship, Poland.

## Abbreviations

The following abbreviations are used in this manuscript:

COPD	chronic obstructive pulmonary disease
EAT-10	Eating Assessment Tool-10 questionnaire
FEES	Flexible Endoscopic Evaluation of Swallowing
PAS	Penetration–Aspiration Scale
SLP	speech language pathologist
VFSS	Videofluoroscopy Swallowing Study
VVST	Volume–Viscosity Screening Test
SSQ	Sydney Swallow Questionnaire
GUSS	Gugging Swallowing Screen
TOR-BSST	Toronto Bedside Swallow Screening Test
DyMUS	Dysphagia In Multiple Sclerosis
PD	Parkinson Disease
MS	Multiple Sclerosis
ALS	Amyotrophic Lateral Sclerosis
MSA	multiple system atrophy
MDPI	Multidisciplinary Digital Publishing Institute
DOAJ	Directory of open access journals
TLA	Three-letter acronym
LD	Linear dichroism
